# ‘*Cand.* Actinochlamydia clariae’ *gen. nov., sp. nov.,* a Unique Intracellular Bacterium Causing Epitheliocystis in Catfish (*Clarias gariepinus*) in Uganda

**DOI:** 10.1371/journal.pone.0066840

**Published:** 2013-06-24

**Authors:** Andreas Steigen, Are Nylund, Egil Karlsbakk, Peter Akoll, Ingrid U. Fiksdal, Stian Nylund, Robinson Odong, Heidrun Plarre, Ronald Semyalo, Cecilie Skår, Kuninori Watanabe

**Affiliations:** 1 Department of Biology, University of Bergen, Bergen, Norway; 2 Institute of Marine Research, Bergen, Norway; 3 Department of Zoology, Makerere University, Kampala, Uganda; 4 Department of Biology, Kampala University, Kampala, Uganda; University of Vienna, Austria

## Abstract

**Background and Objectives:**

Epitheliocystis, caused by bacteria infecting gill epithelial cells in fish, is common among a large range of fish species in both fresh- and seawater. The aquaculture industry considers epitheliocystis an important problem. It affects the welfare of the fish and the resulting gill disease may lead to mortalities. In a culture facility in Kampala, Uganda, juveniles of the African sharptooth catfish (*Clarias gariepinus*) was observed swimming in the surface, sometimes belly up, showing signs of respiratory problems. Histological examination of gill tissues from this fish revealed large amounts of epitheliocysts, and also presence of a few *Ichthyobodo* sp. and *Trichodina* sp.

**Methods and Results:**

Sequencing of the epitheliocystis bacterium 16S rRNA gene shows 86.3% similarity with *Candidatus* Piscichlamydia salmonis causing epitheliocystis in Atlantic salmon (*Salmo salar*). Transmission electron microscopy showed that the morphology of the developmental stages of the bacterium is similar to that of members of the family Chlamydiaceae. The similarity of the bacterium rRNA gene sequences compared with other chlamydia-like bacteria ranged between 80.5% and 86.3%. Inclusions containing this new bacterium have tubules/channels (termed actinae) that are radiating from the inclusion membrane and opening on the cell surface or in neighbouring cells.

**Conclusions:**

Radiation of tubules/channels (actinae) from the inclusion membrane has never been described in any of the other members of Chlamydiales. It seems to be a completely new character and an apomorphy. We propose the name *Candidatus* Actinochlamydia clariae *gen. nov., sp. nov.* (Actinochlamydiaceae *fam. nov.,* order Chlamydiales, phylum Chlamydiae) for this new agent causing epitheliocystis in African sharptooth catfish.

## Introduction

The epitheliocystis condition in fish was first described in 1920 from *Cyprinus carpio* by Plehn [1] as mucophilosis and believed to be caused by a unicellular alga. It was not until 1969 that epitheliocystis was identified as a bacterial infection with a *Bedsonia (Chlamydia)*-like obligate intracellular bacterium producing cysts in the gills of bluegill, *Lepomis macrochirus* [2].

Members of *Chlamydia* were recognised as bacteria and subsequently as important bacterial pathogens in the early 1960ties [3], as members of a new order, Chlamydiales, in 1971 [4] and as a phylum in 2001 [5]. Chlamydia-like bacteria have been identified as pathogens in a large number of phylogenetically very distantly related hosts, including epitheliocystis-causing agents in more than 50 different fish species from fresh and sea water, including sharks and sturgeons [6], [7], [8], [9], [10], [11], [12], [13], [14]. Fish gill epithelial cells infected with *Chlamydiae-*like bacteria show hypertrophy with a large vacuole, called the inclusion, filled with basophilic bacteria. The bacteria are variable in size and morphology and, to a large extent, similar to the characteristic developmental stages described for members of Chlamydiaceae: reticulate bodies (RB), intermediate bodies (IB) and elementary bodies (EB) [15]. The latter is the infective transmission stage of the bacteria. In the epitheliocysts of fish this stage is only present in the larger inclusions.

Little is known about the diversity of the fish-infecting members of the Chlamydiae. The two chlamydia-like bacteria characterized from fish belong to two families, Clavichlamydiaceae and Piscichlamydiaceae [6], [9]. Epitheliocystis agents in fish have also been identified as members of others families like Parachlamydiaceae and Simkaniaceae based on partial 16S sequences [6], [9], [7], [13], [16], [17].

The signature sequence 16S rRNA has only been obtained from a small number of bacteria from epitheliocysts in fish gills. One of these bacteria (*Candidatus* Branchiomonas cysticola), associated with epitheliocystis on the gills of farmed Atlantic salmon in Norway, is not a member of phylum Chlamydiae, but a member of β-proteobacteria [18], [19], [20]. Hence, not all the epitheliocystis agents described from gills of different fish species are necessarily members of Chlamydiales. One member of Chlamydiales, *Candidatus* Renichlamydia lutjani, detected in the blue-striped snapper (*Lutjanus kasmira*), has been suggested to be present in cysts mainly in the kidney and occasionally in the spleen of the host [21]. The authors, however, did not show presence of bacteria in the cysts nor did they locate the bacterium in the host using *in situ* hybridisation.

In September 2011 and March 2012 we collected African sharptooth catfish (*Clarias gariepinus* (Burchell, 1822)) in a fish farm outside Kampala, Uganda, close to the shores of Lake Victoria. The fish showed clear signs of respiratory problems, and histological studies of gill tissues showed heavy infections with epitheliocystis. Transmission electron microscopy studies revealed that a Chlamydia-like agent produced cysts with a unique morphology. The partial 16S rRNA gene showed that the bacterium represents a new lineage among Chlamydiales. This paper gives a morphological and molecular description of the developmental stages and the inclusion of this new and remarkable bacterium. Based on the morphological characteristics and on the criteria given by Everett et al. [22] the bacterium is described as a new species in a new family, Actinochlamydiaceae, within the order Chlamydiales.

## Materials and Methods

### Sampling

African sharptooth catfish (*Clarias gariepinus* (Burchell, 1822)) juveniles, about 10 cm long, were collected from concrete tanks in a fish farm in the Kampala area, Uganda, in late September 2011. The fish were sampled from a population that suffered mortality associated with gill problems. The juvenile fish displayed abnormal swimming behaviour at the pond surface, and some of them where swimming with the belly up. A few older fish, 20–30 cm long, showing no clear signs of disease, were sampled from neighbouring tanks. The fish were alive on arrival in the laboratory. Tissue samples were taken from the gills and other tissues from the juveniles and the older catfishes. These samples were stored in 100% ethanol or in a modified Karnovsky fixative. An additional sampling consisting of newly hatched larvae of *C. gariepinus* (2–3 cm long) and surviving individuals from the same population as that which suffered mortality in September 2011, was carried out in March 2012. Material from the second sampling was stored in 100% ethanol and in buffered formalin. All samples were transported to the University of Bergen, Norway, for histological examination and molecular characterization of possible pathogens associated with the gill tissues.

### Histopathology

The collected tissues (gills, skin and fins) were fixed by immersion in a modified Karnovsky fixative where the distilled water had been replaced by a Ringers solution. The fixative contained 4% sucrose. Before embedding in EMBED-812 (Electron Microscopy Sciences) the tissues were stained/post-fixed in 1% OsO_4_ for 60 minutes. Semi and ultrathin sections were cut on a Reichert-Jung Ultracut E. Semithin sections, 1.0 µm, were stained in toluidine blue. The ultrathin sections (about 30 nm) were stained for 90 minutes in a 2% aqueous uranyl acetate solution and followed by lead citrate. The semi- and ultra sections were used for examination of presence of gill pathogens, their identification and morphological description.

### RNA/DNA Extraction

The extractions of RNA from the gill tissue were done as described by Devold et al. [23]. DNA was extracted from gill tissues using the DNeasy DNA Tissue kit (Qiagen) as recommended by the manufacturer. Elution of the DNA was performed twice in 50 µl 10 mM Tris-HCl pH = 8.5, to increase the overall DNA yield. RNA and DNA were stored at −20°C.

### PCR/real Time PCR

The RNA from the tissues of the catfish was tested by Taqman real time RT PCR for presence of *Candidatus* Piscichlamydia salmonis [24], *Candidatus* Clavichlamydia salmonicola (assay: Ach-F; 5′- AGA ACC TTA CCC AGA TTT GAC ATG T -3′, Ach-probe; 5′- CGT GAC AGC GAT AG AG -3′, Ach- R; 5′- CCT GTC CTT TCG GAA GAC GAT -3′), and *Ichthyobodo* spp. [23]. The real time assays were used as qualitative ± assays and not for quantification. The assays, targeting 16S rRNA gene of *Ca.* P. salmonis and *Ca*. C. salmonicola and 18S rRNA gene of *Ichthyobodo spp,* were run with 2 µl of DNA obtained from gill tissues. Each run consisted of 45 cycles. All samples were negative for presence of *Ca*. P. salmonis and *Ca*. C. salmonicola, while the histological examination revealed presence of epitheliocystis.

DNA was extracted from the gill tissues of the catfish in an attempt to identify the bacteria causing epitheliocystis. The DNA was used to obtain the partial 16S rRNA sequence from a new bacterium (Accession no: JQ480299, JQ480300, JQ480301), and for sequencing an *Ichthyobodo* sp and a *Trichodina* sp. present on the gills. The PCR reaction mixture (50 µl) contained 10×PCR buffer with 1.5 mM MgCl_2_ (Amersham Pharmacia Biotech Inc), 25 mM of each dNTP (Promega), 0.2 µM of each primer (Invitrogen), 1 U Taq DNA polymerase (Amersham Pharmacia Biotech Inc.) and 300 ng DNA.

The *Ichthyobodo* sp. 18S rRNA gene sequence (Accession no: JQ821346) was obtained using primers published by Isaksen et al. [25], while the 18S from the *Trichodina* sp. (Accession no: JQ821348) was obtained by the following primers: Tr-F1∶5′- CTC ATA GTA ACT GAT CGG ATC G –3′and Tr-R1∶5′- AGA AGG TTC ACC AGA TCA CTC –3′. Primers used for amplification of the new chlamydia-like agent found on the gills have already been published (cf. [6], [9]). Amplification was performed in a GeneAmp PCR System 9700 machine (Applied Biosystems) at 95°C for 5 min; 35 cycles of 94°C for 30 seconds, 50°C for 45 seconds, 72°C for 2 min followed by extension at 72°C for 10 min and a short storage at 4°C. PCR products were purified with EZNA PCR cycle pure (Omega Biotech) as prescribed by the manufacturer. All gene sequences obtained in this study have been deposited in the GenBank.

Sequencing was then carried out with ABI PRISM BigDye terminator chemistry (version 2) according to Applied Biosystems (ABI). All sequences were assembled using the Vector NTI Suite 9.0 program (InforMax Inc.).

A Taqman real time PCR assay was designed based on the sequences obtained from the new bacterium on the gills of the African sharptooth catfish. The assay consists of the following primers and probe: ChV-F: 5′- GGG ACY CCG AGA GGR ACC TT –3′, ChV-probe: 5′- TRY GAG CGG CCT GTG –3′, and ChV-R: 5′- ARG CCA TTA CCY TAC CAA CAA GCT –3′. RNA obtained from the gills, skin and fin tissues from the catfish were used for screening with the new assay.

### Construction of Dioxigenin (DIG)-labelled RNA Probes by in vitro Transcription

A 776 nt large fragment of 16S rRNA from a *Ca*. A. clariae (100% nucleotide identity with accession no. JQ480300), obtained from the gills of African sharptooth catfish suffering from gill disease (GD), was amplified using the 16sigF/806R PCR assay as described by Draghi et al. [6]. The construction of DIG-labelled, *Ca.* A. clariae specific RNA probes and subsequent testing of probe specificity by dot blot hybridization was performed as described by Karlsen et al. [9].

### In-Situ Hybridization (ISH)

Ethanol preserved infected gills were rehydrated and then postfixed in 4% paraformaldehyde solution in PBS (pH7.5), and embedded in paraffin according to standard protocols. Series of paraffin sections (3 µm) were cut (Super frost plus slides), some of which were stained with Masson’s HES stain. The tissues for ISH were prepared as follows: the sections were rehydrated after removing paraffin in 2×10 min xylene, down an ethanol gradient (2×100%, 70%, 50%; 2 min steps) to PBS. After rinsing in 0.05 M Tris (pH 7.5), sections were permeabilized by 20 min incubation with proteinase K (5 µg ml^−1^ Tris) at room temperature (RT), followed by washing in Tris. The sections were then treated in triethanolamine (TEA, 0.1 M, pH 8.0) for 10 min, and acetylated for 10 min in TEA (0.1 M) with 0.25% (v/v) acetic anhydride, pH 8.0, and finally washed in PBS. Acetylated sections were prehybridized for 2 h in a humid chamber at RT in hybridization buffer (50% deioinized formamide, 5×standard saline citrate (SSC), 5×Denhardt’s solution (Sigma, USA), 250 mg mly1 tRNA (Roche Molecular Biochemicals), 500 mg ml^−1^ salmon sperm DNA (Roche) and 10% dextran sulphate). Hybridization was performed using 500 ng ml^−1^ DIG-AP cRNA probe in hybridization buffer for 16 h at 65°C in a humid chamber, and followed by washing 30 min in 5×SSC at RT, 15 min in 30% formamide 5×SSC at 65°C, 2×15 min in 0.2×SSC at 65°C, and 5 min in 0.2×SSC at RT. The sections were then washed in RNase buffer (0.01 M Tris, 0.5 M NaCl, 0.005 M EDTA) at 37°C, followed by 25 min incubation in 10 mg ml^−1^ RNase A (20 mg ml^−1^; Promega) at 37°C, washing 5 min in RNase buffer at 37°C, and finally 2×30 min washing in 0.2×SSC at RT. Visualization of DIG-AP was obtained by washing in Buffer 1 (0.1 M Tris, 0.15 M NaCl, pH 7.5) at RT, followed by 1 h incubation in Buffer 2 (Buffer 1 with 1% heat inactivated goat serum) at RT. Sections were then incubated for 16 h at 4°C with sheep anti-DIG-AP antiserum diluted 1∶2000 in Buffer 2. After washing in Buffer 1 and Buffer 3 (0.1 M Tris, 0.1 M NaCl, 0.05 M MgCl2, pH 9.5) at RT, the sections were incubated in Buffer 4 (Buffer 3 with 340 mg ml^−1^ 4-nitro blue tetrazolium chloride, 175 mg ml^−1^ 5-bromo-4-chloro-3- inolyl phosphate-4-toluidine salt, 1 mM Levamisole) between 20–25 min in dark at RT. The color development was stopped by immersion in TEN buffer (0.01 M Tris, 0.001 M EDTA, 0.9% NaCl, pH 8.0) for 10 min, 30 sec in 100% ethanol, and TEN again for 5–10 min. The sections were mounted in TEN with 50% glycerol, and sealed with nailpolish. As controls, adjacent sections were hybridized with a sense riboprobe. Hybridization with sense probes always gave negative results.

### Phylogeny

The sequence data were preliminary identified by GenBank searches done with BLAST (2.0). The Vector NTI Suite software package was used to obtain multiple alignments of nucleotide sequences. To perform pairwise comparisons of the 16S rRNA sequences from the epitheliocystis agent from the catfish, the multiple sequence alignment editor GeneDoc (available at: www.psc.edu/biomed/genedoc) was used for manual adjustment of the sequence alignment. Selected sequences from other members of order Chlamydiales, already available on the EMBL nucleotide database, were included in the comparisons.

Phylogenetic trees were obtained by analyses of the 16S rRNA sequences from selected members of order Chlamydiales. The trees were constructed by maximum likelihood (ML) using TREE-PUZZLE 5.2 (available at: http://www.tree-puzzle.de). The data were analysed using a HKY nucleotide evolution model. The ML tree was bootstrapped in TREE_PUZZLE (50 000 puzzling steps). The bacteria *Lentisphaera araneosa* and *Opitutus terrae* were used as an out-group in the phylogenetic analysis. Phylogenetic trees were drawn using TreeView [26].

### Ethics Statement

Individuals of the catfish *Clarias gariepinus* were collected from fishponds in Uganda. The fish were treated according to the Norwegian Animal Welfare Act (01.01.2010) and the study strictly followed the regulations set by the Norwegian Food Safety Authority. To ameliorate suffering, the collected fish were immediately put into containers with ice-cooled water and taken to the lab. In the lab the lethargic fish were anaesthetised by a blow to the head and killed instantly by decapitation or by severing the brain and cutting off blood transport to the brain. This procedure complies with Norwegian fish welfare regulations.

To carry out research on live animals in Norway one has to have adequate training and a special certificate issued by the Norwegian Government. Professor A. Nylund who participated in and supervised the fieldwork, the collecting, transport to the laboratory, and laboratory treatment of the fish, has this authorisation. All experiments were conducted in accordance with ethical requirements for both Norway and Uganda.

## Results

*Clarias gariepinus*, African sharptooth catfish, were cultured outdoor in concrete tanks close to Lake Victoria on the outskirt of Kampala City. Different generations were kept in neighbouring, separate tanks supplied from the same fresh water source. Wild fish were present in the water source. The larger catfish and the newly hatched fry did not show any clear signs of disease, while the juveniles, about 10 cm in length, showed aberrant behaviour by swimming or resting in the water surface, sometimes swimming with the belly up. The juveniles were dark pigmented and seemed to be suffering from respiratory problems. Mortality rate and the cumulative mortality in the juvenile group are not known.

### Real Time PCR Screening

Real time PCR screening of gill tissues, using the newly designed ChV-assay targeting the 16S DNA from this bacterium, resulted in Ct values ranging from 5.7–11.1 when testing the juveniles, indicating that large amounts of this bacterium was present in the gill tissue. The newly hatched fry, collected in March 2012, were negative for presence of this bacterium and the larger fish from the same sampling period were only slightly positive (Ct values ranging from 22.1 to 33.8). It was not possible to detect this bacterium in the skin or fins from any of the developmental stages of the collected catfish.

Real time PCR screening of the gills from the juveniles showing signs of respiratory problems, showed presence of *Ichthyobodo* sp (Accession no: JQ821346) and microscopic examination of the gill revealed presence of *Trichodina* sp. (Accession no: JQ821348).

### Histology

**Figure 1:**
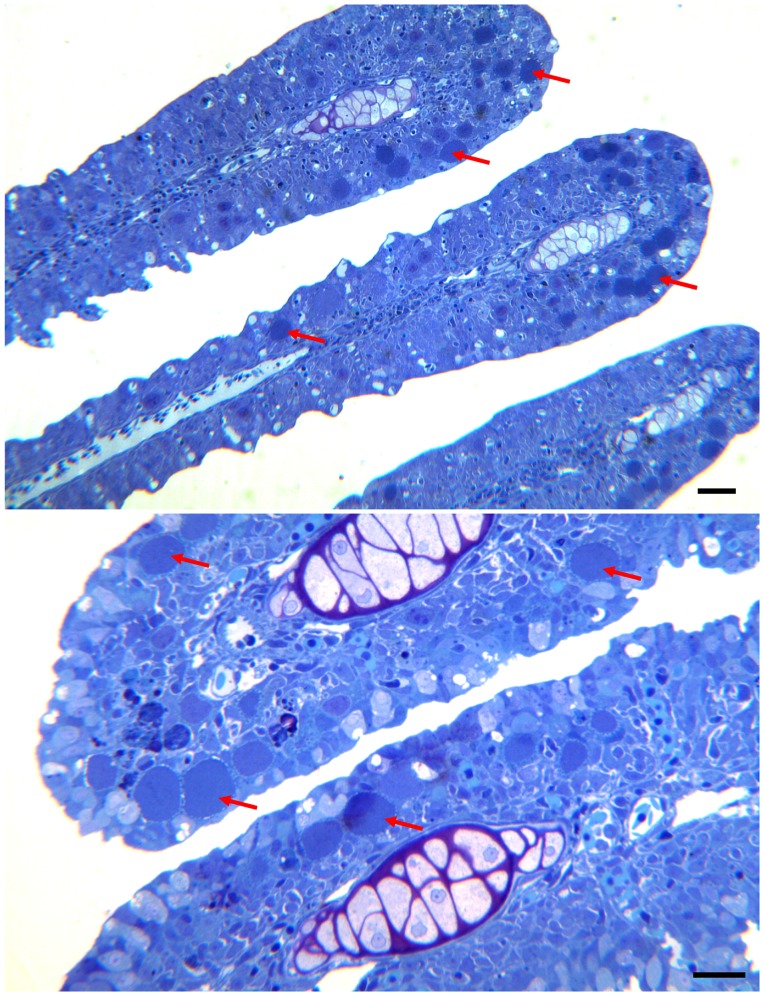
Semi-thin sections of infected primary lamellas from African sharptooth catfish. Pictures of semi-thin sections of the primary gill lamellas from African sharptooth catfish infected with *Candidatus* Actinochlamydia clariae. Arrows point to cysts of variable sizes. The majority of the cysts are located towards the apical part of the primary lamellas. A) Bar = 60.0 µm. B) Bar = 30.0 µm.

**Figure 2:**
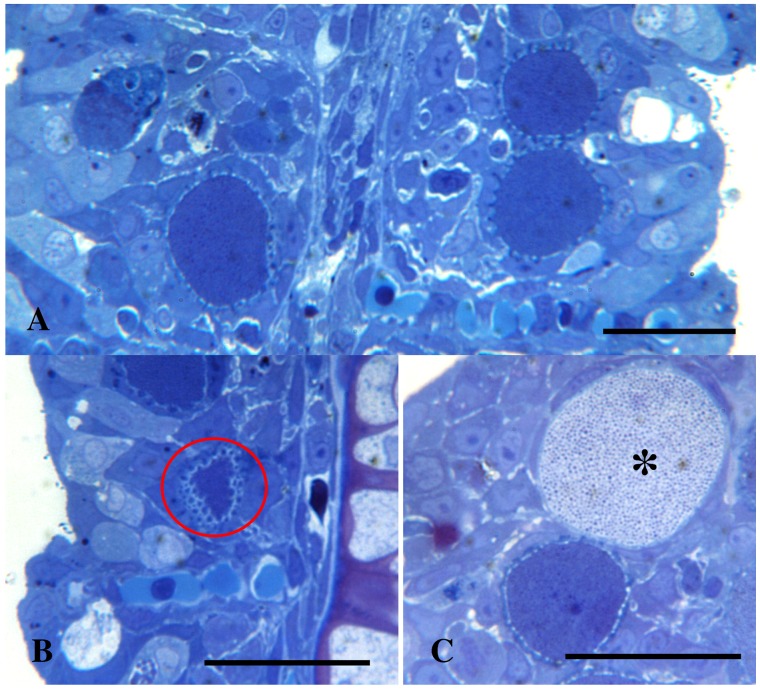
Cysts of *Candidatus* Actinochlamydia clariae. High magnification pictures of cysts containing *Ca*. A. clariae. Figures A) and B) show medium size cysts with tubules/channels radiating from the inclusion giving the cysts its distinct morphology. A) and B) Bars = 30.0 µm. B) A tangential section of the cyst clearly shows that the projections are tubules/channels (ring). C) This figure shows a large cyst, about 30 µm in diameter, containing mostly EBs (asterisk). C) Bar = 30.0 µm.

**Figure 3:**
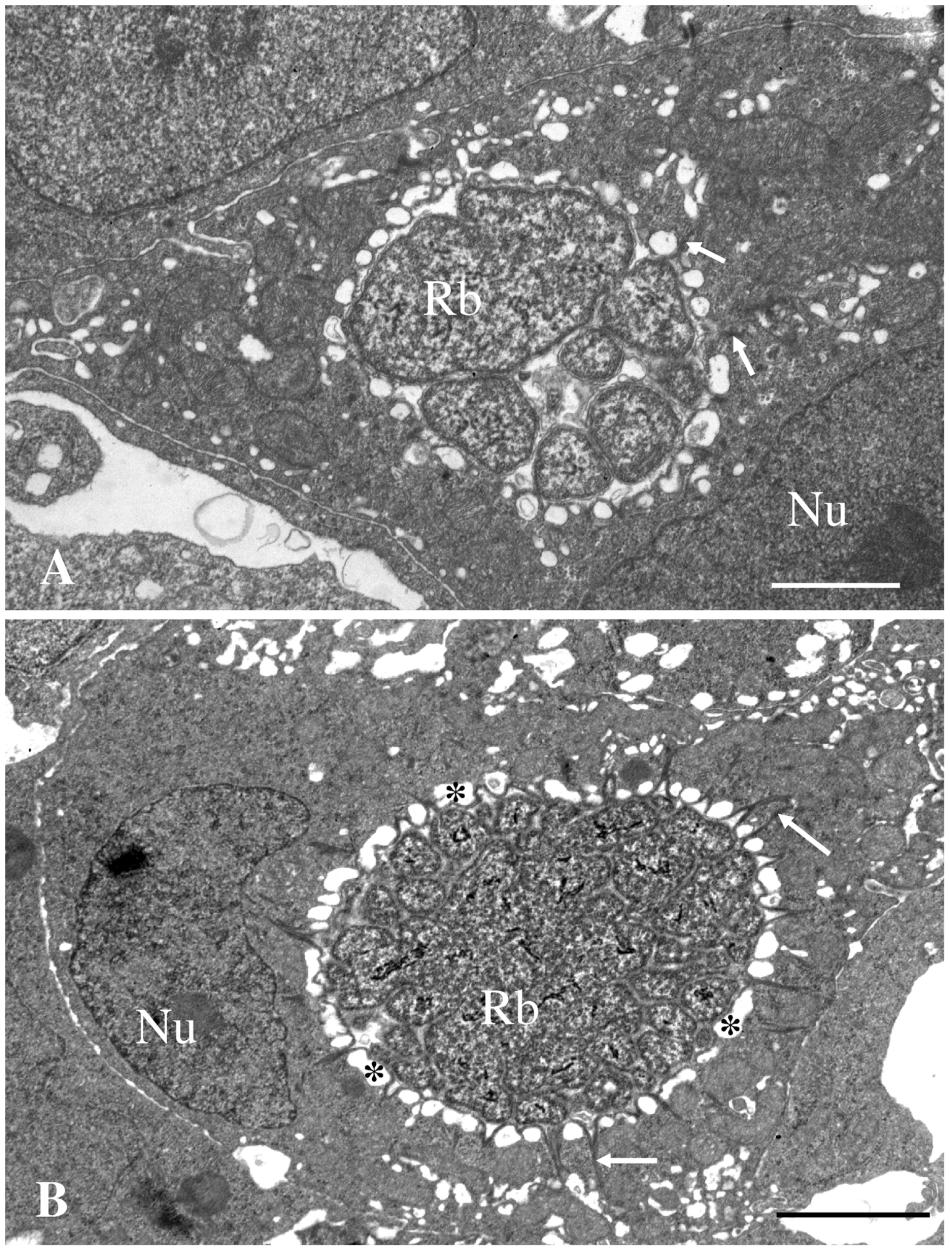
Infected cell with inclusion membrane and RBs. A) Picture of a newly infected cell that contains a few RBs that is surrounded by a thin inclusion membrane. The tubular extensions (arrows) from the inclusion membrane are thin and short. Abundant amounts of mitochondria and small vesicles are present in the cytosol of the host cell close to the inclusion membrane. Bar = 1.0 µm. B) A later stage in the early development of *Ca.* A. clariae. The inclusion contains RBs only, and the tubular extensions (arrows) from the inclusion membrane are longer and more distinct. The inclusion is surrounded by large vesicles (asterisks) and mitochondria. The host cell nucleus (Nu) seems to be slightly distorted. Strands of DNA can be seen in the RBs. Bar = 2.0 µm.

**Figure 4:**
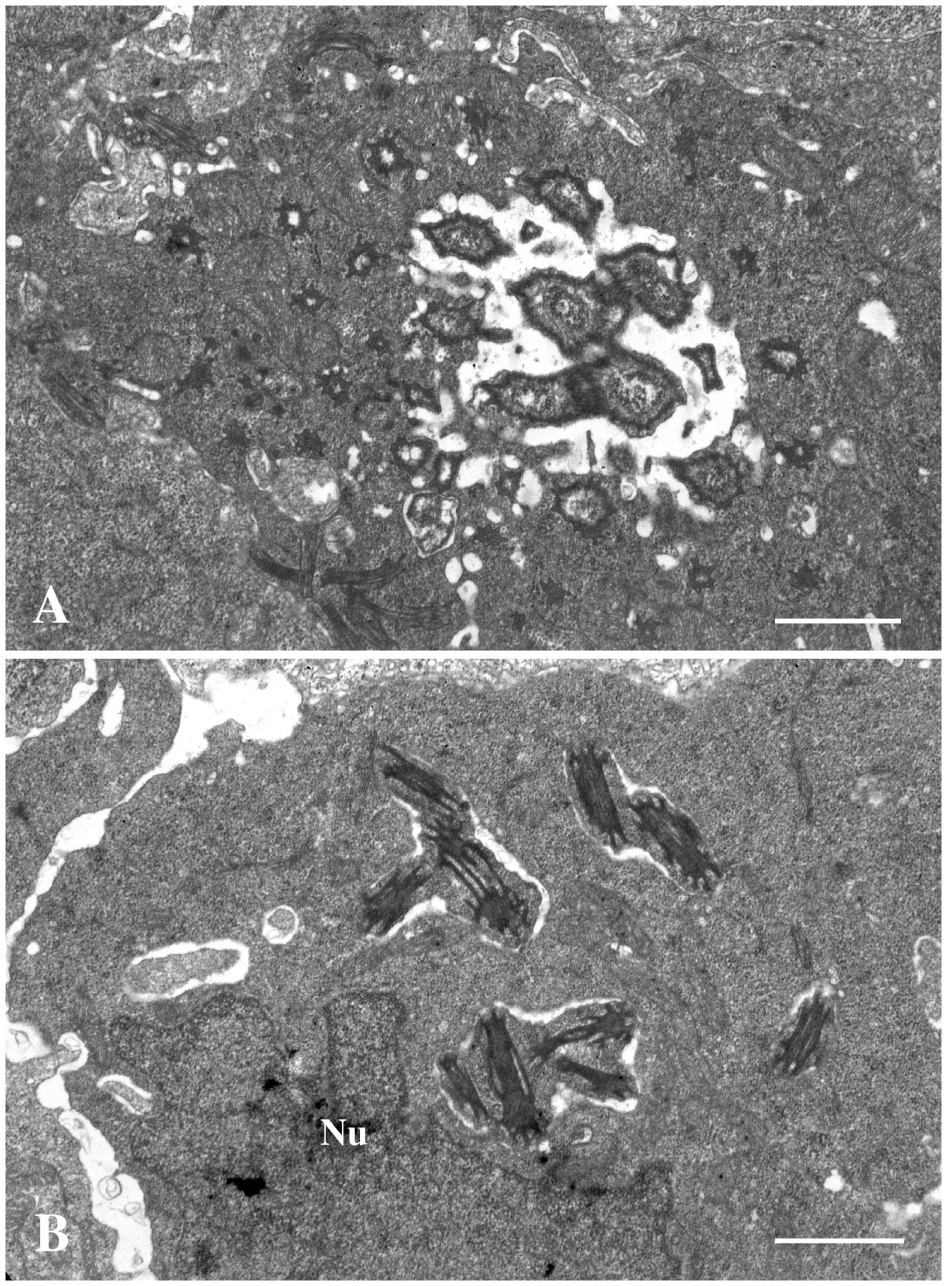
Morphology of tubular extensions. These two figures show the tubules from the inclusion. Picture A) shows transverse sections of the tubules close to the inclusion membrane. The tubules are irregular, star-shaped, with electron lucent material close to the inclusion membrane. Bar = 1.0 µm. B) shows a section through a neighbouring cells showing that the tubules, containing electron dense material, are extending into this cell. Cell nucleus (Nu). Bar = 1.0 µm.

**Figure 5:**
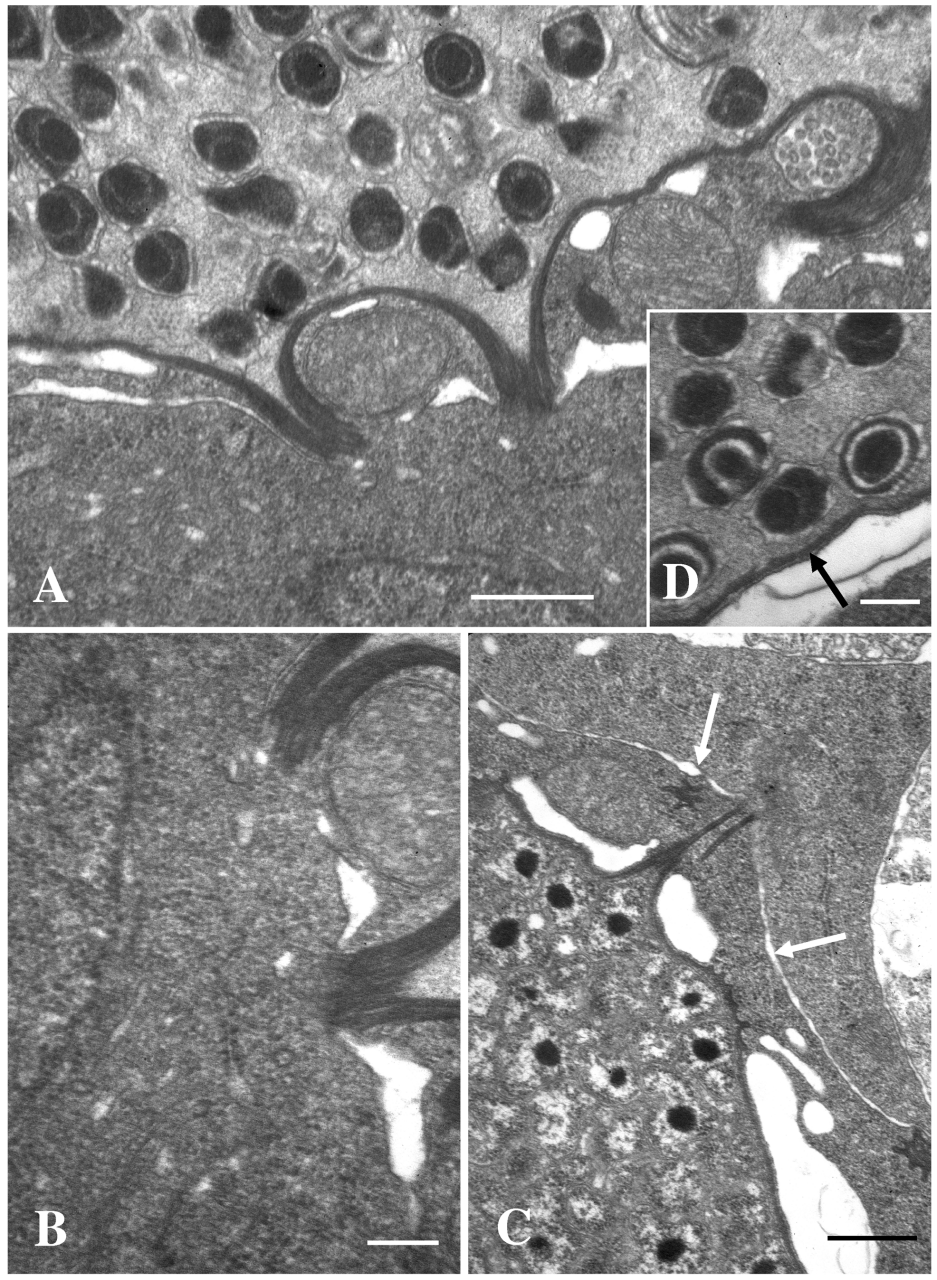
Sections through inclusion membrane. Sections through the membrane of an inclusion containing IBs and EBs. A) Tubular extensions from the inclusion are penetrating into the cytosol of a neighbouring cell. Note that there is no cell membrane separating the tubular opening from the cytosol of the neighbouring cell. Bar = 0.5 µm. B) This is a magnification of the area where two tubules enter the cytosol in figure 5A. Bar = 0.2 µm. C) Tubule from a smaller inclusion penetrating into the cytosol of a neighbouring cell. There seem to be a slight accumulation of fibrils (actin?) in the cytosol where the tubule enters. Note the large vesicles close to the inclusions in infected cells. Cell membrane (arrows). Bar = 0.2 µm. D) This is a section through the inclusion membrane of a cyst containing mainly late IBs and EBs. The inclusion membrane is very thick, probably due to insertion of bacterial proteins (arrow). Bar = 0.5 µm.

The gills of the moribund juveniles showed presence of a high number of hypertrophic cells containing bacteria. This is consistent with epitheliocystis (Figure 1 & 2). Hyperplasia was observed in areas with high numbers of epitheliocysts resulting in interlamellar filling, fusion, and loss of distinct secondary lamellas. Cells infected with the bacterium ranged in size from normal cell size (newly infected cells, probably epithelial cells) to highly enlarged cells with a diameter of about 30 µm. The majority of the epitheliocysts were located towards the tip of the primary lamella and at the bases of the secondary lamellas (Figure 1). Ultrastructural studies revealed that all infected cells (epitheliocysts) contained an inclusion (vacuole) surrounded by an inclusion membrane. A high number of proteins seems to be inserted into the inclusion membrane causing it to increase in thickness as the inclusion grew in size. A host cells nucleus, slightly distorted and located to the periphery of the cell, could be observed in some of the infected cells. The remaining part of the host cell cytoplasm contained mitochondria, ribosomes, and vesicular membrane structures (possibly Golgi vesicles). The cytoplasm was also penetrated by numerous ‘channels’/’tubular structures’ radiating from the inclusion membrane (Figure 3). We refer to these structures as actinae (‘rays’). The actinae usually had longitudinal ridges, giving them a characteristic star-shaped transverse section (Figure 4A). These actinae may open on the host cell surface and may even continue into neighbouring cells opening in the cytosol of these cells (Figure 4 & 5). The actinae, which are extensions from the inclusion membrane, are composed of electron dense material, probably proteins. This material seems to be of the same type with proteins inserted, as that constituting the inclusion membrane. In semi-thin sections the channels/tubules give these epitheliocysts a very distinct morphology with radiations going from the inclusion vacuole (Figure 2). Large cysts, containing small distinct, coccoid bacteria, may open on the gill surface releasing the bacteria to the surrounding water.

### Bacterium Morphology and Developmental Cycle

The inclusions of the infected cells contain polymorphic bacteria with a large variation in size and structure. The different morphs resemble the developmental stages of members of Chlamydiaceae. They can be divided into reticulate bodies (RB) and elementary bodies (EB) with transitional stages (intermediate bodies, IB) between these two. All stages of the bacterium are surrounded by a cell wall and a cytoplasmic membrane (unit membrane).

**Figure 6:**
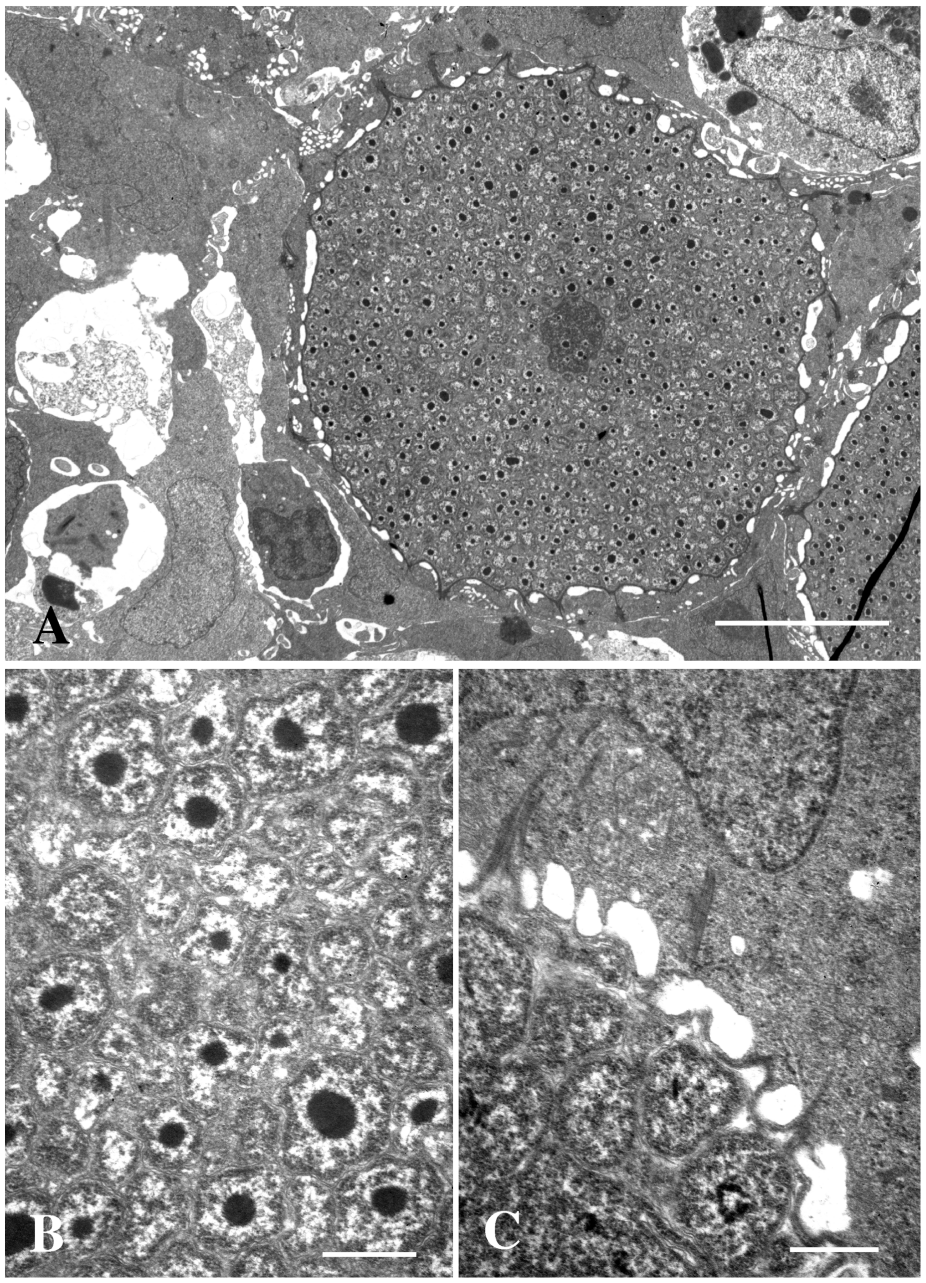
Section through inclusion with IBs. Sections through inclusions containing mainly IBs. The IBs have condensed nucleoids, but the rest of the content of the bacteria is not condensed. A) Bar = 5.0 µm. B) Bar = 0.5 µm. C) Bar = 0.5 µm.

**Figure 7:**
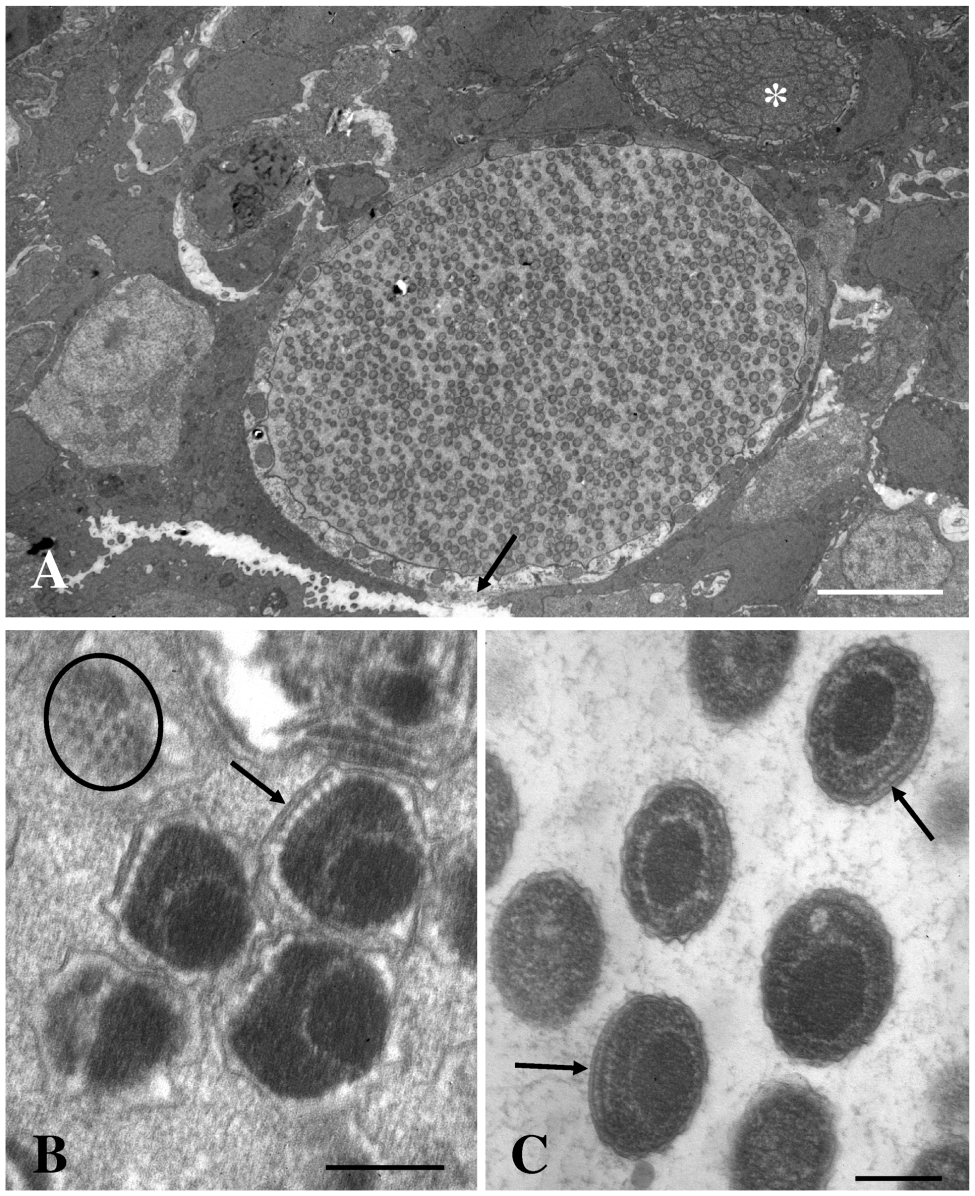
Section through inclusion with EBs. Section through a large inclusion from the gills of *Clarias gariepinus* containing mainly EBs. A) The cyst is beginning to open to the gill surface (arrow), and the host cell cytosol shows signs of degeneration. A neighbouring cell contains an inclusion with RBs only (asterisk). Bar = 5.0 µm. B) Section through EB showing the cap area with associated protein structures (arrow). Tangential section through the cap area (ring) showing the hexagonal arrangement of the proteins. Bar = 0.2 µm. C) Section through free EBs showing the smooth cap areas (arrows) with proteins, an electron dense core (nucleoid), and condensed cytoplasm consisting mainly of ribosomes. Bar = 0.2 µm.

Small inclusions could contain one large bacterium (RB) only, while the larger inclusions contain a high number of bacteria with variable size and morphology. The RB varies in morphology from large spheres to highly pleomorphic shapes with variable size (Figure 3). This developmental stage contains amorphous granular material (ribosome-like) and a few electron dense, irregular, strands (DNA-like). As the number of branches extending from the RBs increases and nucleoids start to appear in the most peripheral part of the branches, parts of the branches divide by fission or budding from the RBs forming smaller but still pleomorphic bacteria with one or several nucleoids. These IBs divide further resulting in more or less spherical/coccoid bacteria about 300 nm in diameter (Figure 6). The spherical bacteria seem to condensate into smaller regular coccoid forms, elementary bodies (EB), with a distinct central spherical nucleoid surrounded by granular material (ribosomes) (Figure 7). The EBs, which are 220–250 nm in diameter, are dominating or sometimes the only morph present in the largest cysts. The EBs all contain a polar ‘cap’ area where the cell membrane and wall are at a fixed distance to each other and where both layers are penetrated by rod-like structures arranged in a hexagonal pattern and protruding from the surface of the bacteria (Figure 7). We have counted up to 25 surface projections on tangential sections of such a cap area.

**Figure 8:**
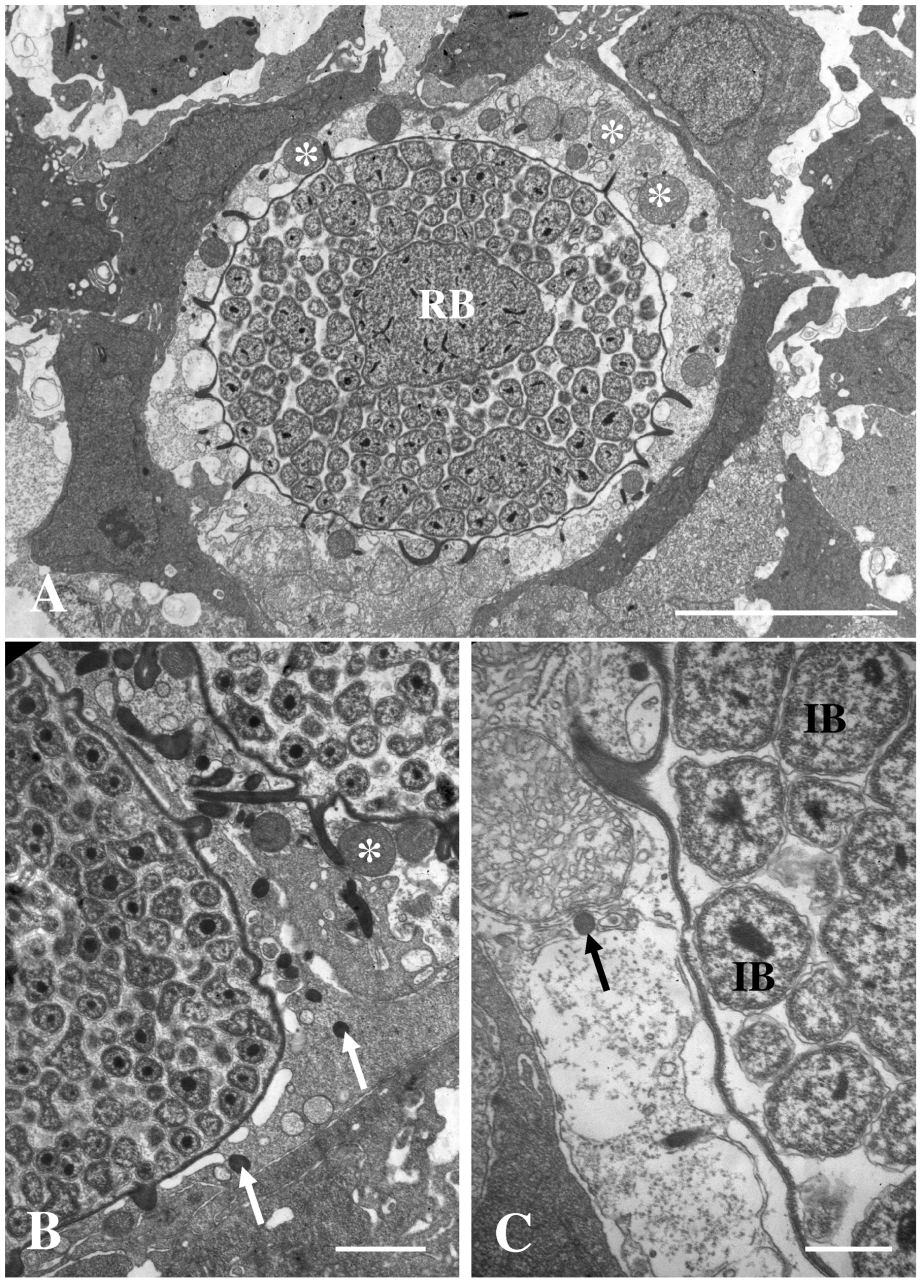
Degenerating epitheliocysts. Pictures of degenerating epitheliocysts from *Clarias gariepinus* that contains inclusion with RBs (RB) and IBs (IB). The host cell cytoplasm loose the normal electron density and the mitochondria increase in size and become spherical (asterisks). The morphology of the tubular extensions from the inclusion changes from the normal irregular, star-shape (in transverse section), to round tubules (arrows). A) Bar = 5.0 µm. B) Bar = 1.0 µm. C) Bar = 0.5 µm.

Degenerating epitheliocysts, containing early developmental stages of the bacterium, were observed in a few sections (Figure 8). The host cell cytoplasm loose the normal electron density and the mitochondria increase in size and become spherical. The morphology of the tubular extensions, from the inclusion in these necrotic cells, changes from the normal irregular, star-shape (in transverse section), to round tubules.

### In situ Hybridization (ISH)

**Figure 9:**
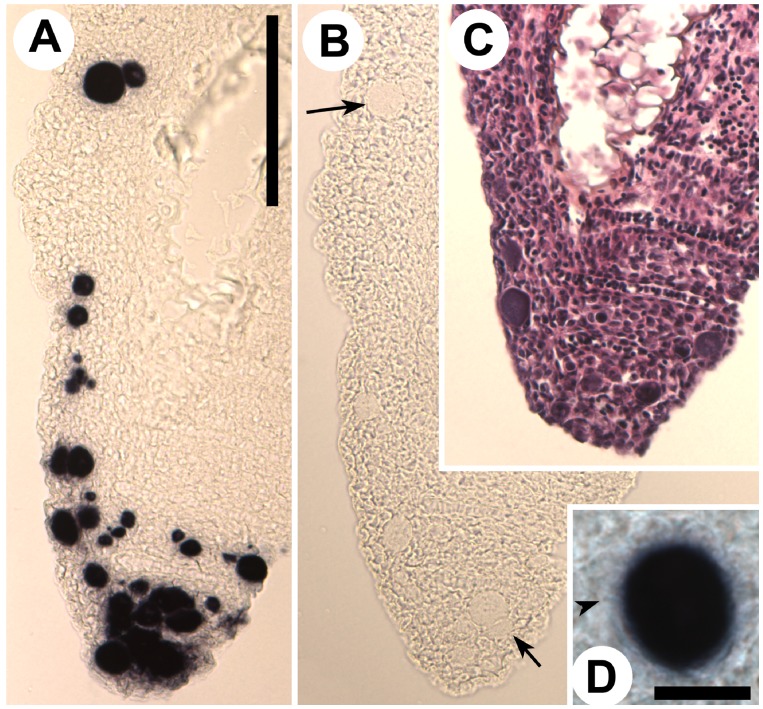
Paraffin sections from gill tissues. The sections of gill tissue from *Clarias gariepinus* have been processed for *in situ* hybridization or stained with HES. A) Primary filament showing dark-blue stained *Candidatus* Actinochlamydia clariae inclusions, stained with antisense DIG-labelled RNA-probe against *Ca.* A. clariae 16S rRNA. Cells with inclusions are particularly frequent at the filament tip. Bar = 100.0 µm. B) Same primary filament stained with a sense probe, demonstrating absence of staining in the inclusions (examples indicated by arrows). Bar = 100.0 µm. C) HES stained section of the same filament tip. Bar = 100.0 µm. D) Magnification of an IHC stained inclusion, where the actiniae are discernible (arrowhead). Bar 10.0 = µm.

The epitheliocystis inclusions in the gill epithelium of African sharptooth catfish were specifically labelled with the 776 nt antisense riboprobe that was transcribed from 16S rRNA (Accession no: JQ480300) amplified from gills of this fish (Figure 9). The ISH positive infected individuals were among those studied ultrastructurally (see above). Inclusions in adjacent histological sections that were incubated with the sense probe (776 nt) were not labelled.

## Discussion

### Phylogeny

**Figure 10:**
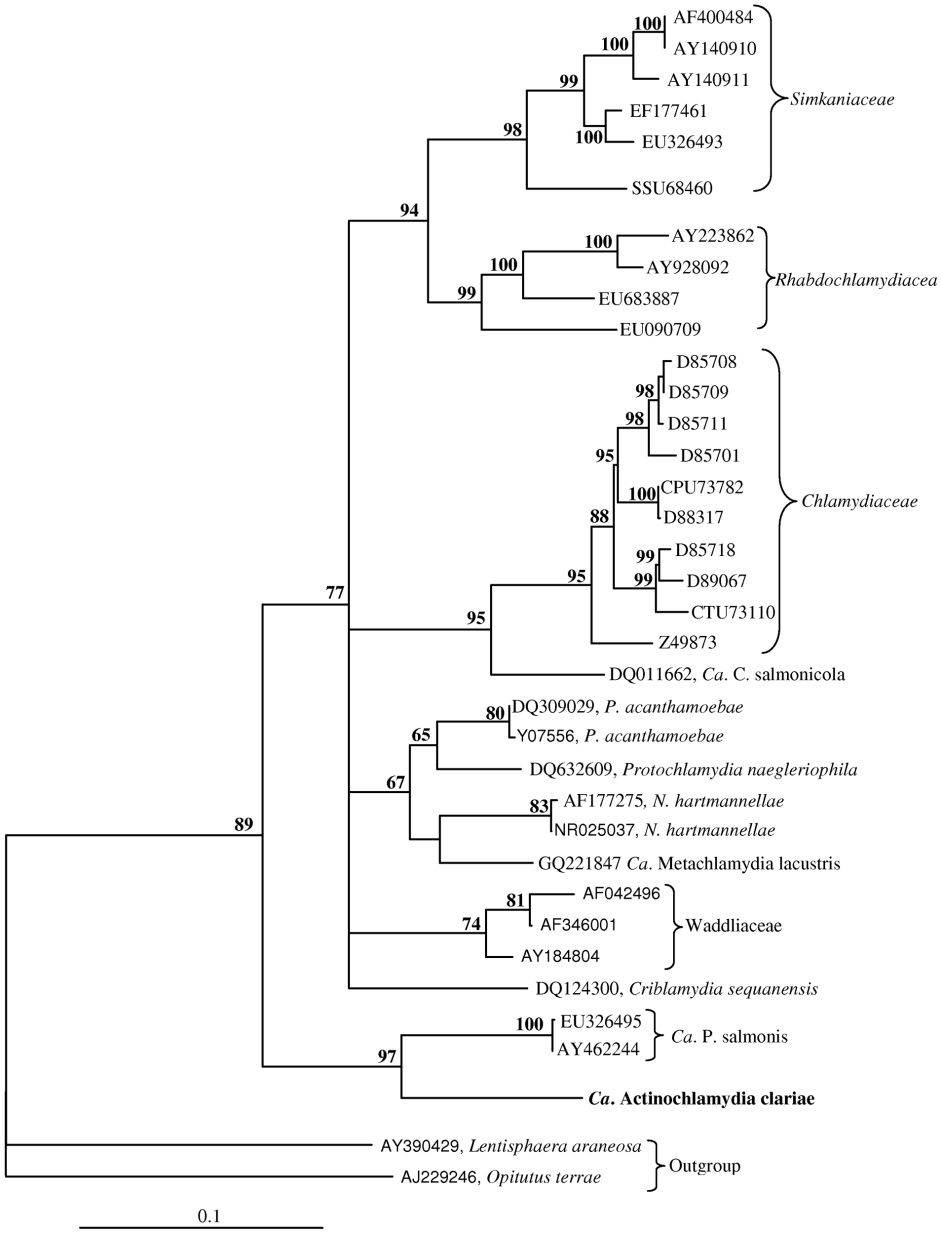
Phylogenetic tree. The phylogenetic tree shows the relationship between *Candidatus* Actinochlamydia clariae from African sharptooth catfish and selected members of other families within the order Chlamydiales. The best-fitting nucleotide substitution model was used during maximum likelihood analysis and the tree was bootstrapped (50 000 quartet puzzling steps) in TREE_PUZZLE. The scale bar shows the number of nucleotide substitutions as a proportion of branch lengths.

The relationship of this new member of the Chlamydiales was examined by phylogenetic analysis using a partial sequence (1280 nt) from the 16S rRNA gene. Members from all families within Chlamydiales were included in the analysis (Figure 10). This new bacterium groups together with *Candidatus* Piscichlamydia salmonis as an early branch within Chlamydiales. However, it differs significantly from *Ca*. P. salmonis showing a sequence similarity of 86.3% when comparing 1428 nucleotides in the 16S rRNA gene. The sequence similarity between the new chlamydiaceae and other members of Chlamydiales, range from 80.5% (*Waddlia chondrophila*) to 83.5% (*Ca*. Metachlamydia lacustris) (Table 1).

**Table 1:** Nucleotide similarity of *Ca*. Actinochlamydia clariae (Accession nos: JQ480299, JQ480300, JQ480301) compared to other members of the order Chlamydiales.

Family - Species	Nucleotides (N = )	% similarity	Accession no
Piscichlamydiaceae			
*Ca*. Piscichlamydia salmonis	1428	**86.3**	AY462244
Chlamydiaceae			
*Chlamydophila pneumoniae*	1405	82.1	Z49873
*Chlamydia suis*	1421	82.3	CTU73110
Clavichlamydiaceae			
*Ca*. Clavichlamydia salmonis	1419	81.8	DQ011662
Simkaniaceae			
*Simkania negevensis*	1424	81.9	U68460
*Ca*. Xenochlamydia salmonis	1340	82.4	EU326493
Rhabdochlamydiaceae			
*Rhabdochlamydia crassificans*	1420	82.3	AY928092
Parachlamydiaceae			
*Parachlamydia acanthamoebae*	1416	83.4	Y07556
*Neochlamydia hartmanellae*	1417	82.4	AF177275
*Ca.* Metachlamydia lacustris	1353	83.5	GQ221847
Waddliaceae			
*Waddlia chondrophila*	1147	**80.5**	AF042496
Criblamydiaceae			
*Criblamydia sequanensis*	1418	82.7	DQ124300

The closest relative sharing a common ancestor with this new member of order Chlamydiales described in the present paper, ‘*Candidatus* Actinochlamydia clariae nov.sp.’, is *Candidatus* Piscichlamydia salmonis characterized by Draghi et al. [6]. The morphology of *Ca*. P. salmonis has been described in earlier papers on epitheliocystis in salmonids [12], [27], and later studies have shown that this is a common bacterium on gills of salmonids in fresh and sea water [14], [17], [28]. However, the morphology of the developmental stages of *Ca.* A. clariae only shows limited similarities to that of *Ca*. P. salmonis [6], [12], [14], [28]. One feature that is shared by both species is the ‘cap’ structure seen in the IBs and EBs (cf. Figure 6B, Nylund et al. [12]), but proteins inserted in the inclusion membrane in this area are less visible in *Ca.* P. salmonis compared to *Ca*. A. clariae. The cysts formed by the two bacteria are also very different. The inclusion membrane surrounding *Ca*. A. clariae vacuoles is very thick, possibly due to insertions of proteins, while the inclusion membrane surrounding developmental stages of *Ca*. P. salmonis is a thin unit membrane with no visible increase in thickness. Budding of vesicles into the inclusion can be observed in the latter. This was not observed in the inclusions of cells infected with *Ca*. A. clariae. The bacteria-containing vacuoles in cells infected with *Ca*. P. salmonis lacks the distinct tubules/channels (‘actinae’) radiating from the inclusions seen in cells infected with *Ca*. A. clariae. Radiation of tubules/channels from the inclusion in infected host cells has never been observed among any of the other members of Chlamydiales. It seems to be a unique character and thus an apomorphy for *Ca*. A. clariae.

The *Ca*. A. clariae inclusions in gill cells of the African sharptooth catfish is distinct from all previous descriptions of host cell inclusions for other members of Chlamydiales [6], [8], [9], [12], [27], [29], [30], [31], [32], [33], [34], [35], [36], [37], [38]. The tubules extending from the inclusions into the host cell cytosol and into neighbouring cells are unique features, which call for an explanation of function. Little is known about the function of the inclusion except for members of family Chlamydiaceae. In some members of this family a type III secretion (T3S) system has been identified and described, which mediates the translocation of bacterial toxins to the cytosol of infected cells [39]. The chlamydial T3S machines span both membranes of the bacterium and the T3S needle can penetrate the inclusion membrane facilitating translocation of secreted effectors across this membrane [39]. The excreted toxins may alter the host cell cytoskeleton, repress or activate apoptosis, or disrupt host transcriptional regulation. The effector proteins are either secreted into the inclusion lumen, deposited in the inclusion membrane, or they are translocated directly to the cytosol of the host cell. The translocated actin-recruiting phosphoprotein (Tarp) is one of the proteins that are translocated through the T3S system. This protein is involved in the recruitment of actin and believed to be ‘preloaded’ in the T3S needle of the EB so that it can mediate early cytoskeletal changes during internalization [40]. Another T3S effector protein is the inclusion membrane protein A (IncA) located on the outer face of the inclusion membrane (cf. Peters et al. [39]). This protein is involved in homeotypic fusion when multiple inclusions occur in a host cell. IncA may also form long fibres extending from the inclusions. Suchland et al. [41] hypothesised that these fibres could be used to create a second intracellular environment and to transport chlamydia to that environment. If this hypothesis is correct then the inclusion membrane is not only a barrier and interface between the bacteria and the host cell cytoplasm, but also an active participant in developing an expanding environment for the developing RBs. It has been shown that inclusion proteins, including IncA, are not specific for members of the Chlamydiaceae, but are also present in distantly related chlamydial groups [42]. The membrane in the tubules extending from the inclusions in host cells infected with *Ca*. A. clariae is morphological identical to that of the inclusion itself. This suggests that it is of the same origin/type as the inclusion membrane and with the same proteins inserted. Based on existing knowledge of the Inc proteins the generation of such tubules may be understood as a result of the action of Inc proteins similar to those described from *Chlamydia* species. Hence, these tubules could be part of a strategy for expanding the environment for the developing RBs of *Ca*. A. clariae by penetrating into neighbouring cells and thereby colonizing these cells. This could explain the large resulting cysts observed on the gills of *Clarias gariepinus*. This interpretation is based on morphological observations only. Further work is necessary to corroborate this hypothesis before concluding that this may represent a new mechanism for growth of chlamydia inclusions.

Chlamydiae obtain amino acids and nucleotides from host pools, but the mechanisms are not fully understood. In the closest relative to *Ca*. A. clariae, *Ca*. P. salmonis, and also in the more distantly related fish pathogen, *Ca*. C. salmonicola, budding of vesicles into the inclusion is frequently observed during bacteria development [6], [9], [27]. Both species have a thin-walled inclusion wall. The inclusions for *Ca*. A. clariae are thick-walled and at no developmental stage could we observe vesicles budding into the inclusion or fusion of such vesicles with the inclusion membrane. The actinae of *Ca*. A. clariae may connect the inclusion with the host cell cytosol, host cell surface and/or even with the cytosol of neighbouring cells. Cross sections of the actinae show that they are tubules (cf. Figure 4A), indicating that they might have a transport function. These actinae could be important for the expansion of environment for the developing bacteria, but they may also function as channels allowing uptake of low-molecular-weight molecules (e.g. amino acids, nucleotides) for bacterial growth. But this remains to be verified.

The EB is the infective stage of Chlamydiaceae. A crucial step in the entry into host cells is attachment to the host cell membrane. This is mediated by components on the outside of the chlamydia cell envelope. Among the proteins shown to be of importance for attachment and entry into the eukaryotic host cell are the outer membrane protein B (OmcB), the major outer membrane protein (MOMP), and Tarp [39], [43], [44], [45], [46]. The attachment site on the EB is believed to contain a T3S system arranged in a hexagonal array giving a distinct character to this site. Morphological description of these attachments sites on the EBs and attachment of these to hosts cells have been published in several studies [47], [48], [49], [50], [51], [52]. A similar morphology of the EBs can also be seen in the surface cap area of the EB from *Ca*. A. clariae. Based on morphology and the fact that this system is also present in other members of Chlamydiales, it suggests that the cap area of the EBs from *Ca*. A. claria represent the attachment site for the infective stage of this species, thus facilitating internalization in susceptible host cells.

After submission of the present work, Stride at al. [53] published the characterisation of a new species ‘*Candidatus* Parilichlamydia carangidicola’ from yellowtail kingfish, *Seriola lalandi* from South Australia and proposed a new family ‘*Candidatus* Parilichlamydiaceae’. They used a different processing procedure for histology and transmission electron microscopy. Consequently the figures presented in their paper are difficult to compare to the structure of the inclusion and development stages observed in our study. The partial 16S rRNA gene sequence similarity based on 1103 nt (Accession no: JQ673516) is about 92%. When more information is available, the two bacteria may be shown to belong to the same family.

### Description of Actinochlamydiaceae fam.nov

Actinochlamydiaceae (M.L. fem. n. Actinochlamydia, type genus of the family; -aceae ending to denote a family). The family Actinochlamydiaceae currently comprises but a single genus, the type genus Actinochlamydia. Strains with rRNA gene sequences that are ≥90% identical to the ribosomal genes of *Candidatus* Actinochlamydia clariae sequences (JQ480299, JQ480300, JQ480301), should be considered belonging to family Actinochlamydiaceae (cf. Everett et al. [22]). The Actinochlamydiaceae belongs to the order Chlamydiales. It forms a sister taxon to the Piscichlamydiaceae and Chlamydiaceae with a chlamydia-like cycle of replication and with ribosomal genes that are 80–90% identical to ribosomal genes in the Piscichlamydiaceae and Chlamydiaceae.

### Description of Actinochlamydia gen. nov

Actinochlamydia (Gr. n. *aktis -inos*, a ray, M.L. fem. n. *Chlamydia* taxonomic name of bacterial genus). Actinochlamydia fem. n. *Chlamydia* with rays, referring to the distinct tubular structures radiating from the inclusions.

At present the description of Actinochlamydia corresponds to the description of the family Actinochlamydiaceae. Members of the genus show a thickening of the inclusion membrane, with ray-like structures radiating from the inclusion. New members of this genus should have >95% rRNA gene sequence identity with *Ca*. A. clariae sequences (JQ480299, JQ480300, JQ480301).

### Description of *Candidatus* A. clariae sp. nov

*Candidatus* Actinochlamydia clariae [clari’ae M.L. gen. sing. of Clarias, generic name for the African sharptooth catfish; of (living in) members of the genus *Clarias*].

The provisional taxon *Ca.* A. clariae contains an intracellular bacterium within membrane-bound vacuoles (inclusions) in the cytoplasm of gill cells of the African sharptooth catfish, *Clarias gariepinus*. The bacterium has a number of developmental stages similar to those of members of Chlamydiaceae and exhibit a chlamydia-like developmental cycle. The developmental stages include pleomorphic RBs of varying size, IBs ranging from pleomorphic, branching, morphs to coccoid shapes, and slightly oval EBs ranging between 220 to 250 nm in diameter. All stages are surrounded by a cell wall and a cytoplasmic membrane. The EB’s possess a polar ‘cap’ area where the cell membrane is penetrated by rod-like structures arranged in a hexagonal pattern.

The bacterium is non-motile and Gram-negative. The *Ca*. A. clariae 16S rDNA sequence (Accession nos: JQ480299, JQ480300, JQ480301) is 18.0–17.6% different from the 16S rDNA of Chlamydiaceae, fitting the 80–90% identity range that makes this organism a member of Chlamydiales, but not a member of the family Chlamydiaceae. The sequence similarity between *Ca*. A. clariae 16S rDNA and *Ca*. Piscichlamydia salmonis is 86.3%, and less with other members of Chlamydiales (Table 1). For a newly identified strain to be described as a new member of *Ca*. A. clariae a nearly full-length rDNA sequence of the new strain may only differ from *Ca*. A. clariae 16S rDNA by <3% (≥97% similarity).

## Acknowledgments

The authors want to thank all personnel helping us during the sampling programme at fish farms in Uganda in 2011 and 2012. We also thank Anne-Cathrine B. Einen at the Institute of Marine Research, Bergen, for help with the in-situ hybridization.
